# Initial Views and Experiences of Vaping in Prisons: A Qualitative Study With People in Custody Preparing for the Imminent Implementation of Scotland’s Prison Smokefree Policy

**DOI:** 10.1093/ntr/ntaa088

**Published:** 2020-05-24

**Authors:** Ashley Brown, Rachel O’Donnell, Douglas Eadie, Richard Purves, Helen Sweeting, Allison Ford, Linda Bauld, Kate Hunt

**Affiliations:** 1 Institute for Social Marketing and Health, Faculty of Health Sciences and Sport, University of Stirling, Stirling, UK; 2 MRC/CSO Social and Public Health Sciences Unit, University of Glasgow, Glasgow, UK; 3 Usher Institute and SPECTRUM Consortium, College of Medicine and Veterinary Medicine, University of Edinburgh, Edinburgh, UK

## Abstract

**Introduction:**

Scotland is one of the few countries in which e-cigarettes were available in prisons *before* the introduction of a comprehensive national smokefree policy, to assist in its implementation. This qualitative study explores the initial views and experiences of vaping in this specific context, from the perspective of people in custody (prisoners).

**Aims and Methods:**

Twenty-eight people in custody were interviewed approximately 1–2 months *after* rechargeable e-cigarettes were made available in prisons and 2–5 weeks *before* implementation of a smokefree policy. Data were thematically analyzed to identify the range and diversity of views and experiences.

**Results:**

Participants expressed support for e-cigarettes in preparation for the smokefree policy, describing their symbolic and practical value in this context. Uptake of vaping was strongly influenced by the need for participants to manage without tobacco in the near future. Participants evaluated their initial vaping experiences, either positively or negatively, in relation to the utility of e-cigarettes for mandated smoking abstinence and in providing satisfaction, pleasure, and novelty. Participant views on several issues related to e-cigarette use, both specific to the prison population (product choice and cost) and more generally (safety and long-term use), are explored.

**Conclusions:**

Our findings suggest possible benefits of e-cigarettes as one means of supporting smokefree policy in a population with many smokers. They also point to potential challenges posed by vaping in prisons and smokefree settings caring for similar populations. There is a need for ongoing measures to maximize the health benefits of smokefree settings and for further research on vaping in situations of enforced abstinence.

**Implications:**

To our knowledge, no published studies have explored views and experiences of vaping in prison, when rechargeable vapes were new and the removal of tobacco was imminent. The results can inform tobacco control policy choices, planning and implementation in prisons and similar settings. In prison systems that permitting vaping, it is important that other measures (eg, information campaigns and nicotine dependence services) are implemented concurrently to minimize potential risks to the health or personal finances of people in custody.

## Introduction

People in custody (prisoners) experience substantially poorer health than the general population,^1,2^ in part because of the high prevalence of tobacco smoking (henceforth “smoking”) among people in custody^3^: smoking prevalence among those in custody in Scotland (72%) in 2015 was three to four times higher than in the general population.^4^ Secondhand smoke (SHS) is also associated with increased risk of all-cause mortality and death from vascular disease.^5^ Several countries (eg, Canada, New Zealand, England, and Wales) have introduced smokefree policies, to reduce the burden of tobacco on people in custody, and staff exposed to SHS at work. Scotland’s prisons simultaneously became smokefree in 2018; e-cigarettes (disposable and rechargeable with prefilled e-liquids) became available in all prisons *before* tobacco sales ceased, informed by expert consensus as available at that time, that vaping is less harmful than smoking tobacco.^6–8^

There has been much discussion about the balance of benefits and risks of e-cigarette use (hereafter, “vaping”) in public health circles, some focusing specifically on its potential place in smokefree prisons.^9,10^ Arguments for e-cigarettes in prison^11^ relate to their potential to support individuals to stop smoking or cope without tobacco (based on evidence from community settings^12^) and to support prison services through challenging organizational change. It has been suggested e-cigarettes may help to minimize negative organizational consequences associated with prison smokefree policies (eg, aggression/violence and displacement use of other substances).^13^ Possible risks of allowing e-cigarettes^9,10^ in prison include their potential to be repurposed to charge illicit mobile phones or facilitate illicit drug taking, hazards of open-system e-cigarettes, uncertainties surrounding their role in maintaining nicotine dependence, and long-term health effects.

To our knowledge, no published studies have explored views and experiences of vaping in prison. Research on this topic can inform tobacco control policy in prisons and similar settings and wider discussions about whether e-cigarettes support or inhibit reductions in tobacco-related harms, particularly in vulnerable and heavy smoking groups. We have reported Scottish Prison Service (SPS) staff’s opinions relating to the *hypothetical* availability of e-cigarettes in prisons,^14^ using data collected *before* it was known that e-cigarettes would be sold in prisons or that smokefree policy would be introduced. This paper presents findings from interviews with people in custody, conducted immediately after the introduction of rechargeable e-cigarettes into Scotland’s prisons in the 2–5 weeks before the implementation of smokefree policy on November 30, 2018.

## Methods

Qualitative interviews were conducted with people in custody before the introduction of the smokefree policy in Scottish prisons. The study was approved by the SPS Research Access and Ethics Committee and the University of Stirling’s General University Ethics Panel (GUEP 497).

### Sampling and Recruitment

Interviews were conducted in six prisons in Scotland, selected in consultation with the SPS to represent a range of prisoners and prison environments. Participants were recruited through a staff point of contact for reasons of logistics, privacy, and participant/interviewer safety. Each point of contact was asked to select a mix of individuals who had used rechargeable e-cigarettes in prison. The sample for this analysis comprised 28 participants: 22 men and six women; three remanded and 25 convicted individuals, including eight serving short sentences (up to <4 years), 14 long sentences (≥4 years), and three whose length of sentence was not reported. Target sample sizes were set in advance of data collection, informed by our judgments about what size (and composition) of the sample might provide a sufficient account of the research topic, and pragmatic considerations.

### Data Collection

Qualitative interviews were chosen as they enable an in-depth exploration of people’s experiences in one-to-one interactions. Interviews (average length ~40 min) were conducted between October 24 and November 16, 2018 by five research team members (AB, RO, KH, DE, and RP). Interviews were carried out with only the interviewer and participant present to protect privacy, although in one prison a joint interview was conducted at the request of two participants. Consent procedures sought to balance legal and ethical requirements and the literacy and learning needs of the population. Researchers provided participants with verbal and written information about the study, emphasizing in the private setting of the interview that participation was entirely voluntary. If the participant wished to proceed, their consent was audio recorded or provided in writing on a case-by-case basis. The topic guide covered: smoking and vaping history, views and experiences of vaping outside of prison, opinions about e-cigarettes and prison smokefree policy, early experiences of vaping in the prison context, and views on the benefits and challenges of allowing people in custody to vape. Topic guides were used in the in-depth interviews^15^; researchers formulated questions using their own words (often responding to issues raised by participants), probed for more detail and adjusted the topic order as appropriate, and encouraged participants to raise anything which they thought was important, including any recommendations for improvement.

### Analysis

With participant permission, interviews were audio-recorded and transcribed. Transcripts were de-identified before analysis. Data were thematically analyzed using the framework approach.^16^ This involved development of a “thematic framework,” informed by the literature, research questions, and reading the transcripts. A framework grid was constructed using Nvivo 12 software and data summaries (including hyperlinks to raw data) were written (by AB, RO, DE, RP, HS, and AF) in the relevant cell. AB and RO conducted detailed thematic analysis iterating between the framework grid and the transcripts, carefully examining material to identify the range and diversity of responses in relation to topics and creating themes and subthemes. Themes were refined over multiple iterations based on reexamining data, critical reflection, and research team discussion. These strategies also facilitated reflexivity. The final interpretation of the data was agreed by all authors.

Quotations, illustrating the diversity of perspectives across participants and prisons, are included, with their smoking/vaping status (“exclusive-vaper,” “dual-user,” “former-vaper”), custodial status (on remand [R], convicted on a short [ST] or longer term [LT] sentence), and prison code (01-06, randomly allocated to prisons, specifically for this paper, to protect anonymity).

### Context

Several features of the prison setting are likely to have an important bearing on e-cigarette use. Prison wages are relatively low (estimated earnings from 2013 for people in custody in Scotland were £5–12 per week^17^). Additional funds can be provided by family and friends, although not everyone receives this support. People in custody are only permitted to purchase and use e-cigarette products (and other items) that have been selected locally from a list of nationally approved items; and upper limits on weekly spending vary by untried or convicted status and privilege level.^18,19^ At the time of the interviews, people in custody were still permitted to buy and smoke tobacco and two closed-system rechargeable e-cigarettes, selected by the SPS, had recently gone on sale in the canteen (prison shop). E-cigarette “starter packs” were offered free of charge to eligible declared adult smokers who would be in custody on November 30th as a transitional measure. Starter packs included a rechargeable e-cigarette, charger, and three 18 mg/mL tobacco-flavor e-liquids. All subsequent products had to be purchased from the prison shop, where a starter pack (January–June 2019) and one brand of rechargeable e-cigarettes (September 2018–April 2019) were sold at a discounted price for a limited time (Table 1). To our knowledge, there was no variation in the choice of rechargeable e-cigarette products which could be purchased in prison at the time of the interviews. Single-use e-cigarettes had been on sale in the canteen from early 2018. (Unless otherwise stated, “e-cigarettes” hereafter refers to rechargeable e-cigarettes, as single-use e-cigarettes were seldom used.) Conventional stop smoking aids (ie, nicotine replacement therapy [NRT] and varenicline) and/or behavioral support were available in all Scottish prisons via national smoking cessation services.

**Table 1.** E-cigarette Products on Sale in Scottish Prisons at Time of Interviews (October–November 2018)

E-cigarette product	Description	Price discount applied for a limited time?
Rechargeable e-cigarette starter pack	Rechargeable e-cigarette (*Brand A*), charger plug, and three tobacco flavor e-liquids (18 mg/mL)	Yes
Rechargeable e-cigarette (*Brand A*)	Rechargeable e-cigarette taking prefilled e-liquids	No
E-liquids for Brand A	Strawberry 12 mg/mL Berry mint 12 mg/mL Menthol 12 mg/mL Tobacco 18 mg/mL	No
Rechargeable e-cigarette (*Brand B*)	Rechargeable e-cigarette taking prefilled e-liquids	Yes
E-liquids for Brand B	Blackcurrant 18 mg/mL Strawberry 18 mg/mL	No
Single-use e-cigarette	Various brands	Yes

Prisons operate a program of activities and prison population movement in line with prison rules. These specify that smoking (pre-ban) and vaping (post-ban) are not permitted outside of designated rooms (cells) and outdoor areas. In 2018/2019, the average time spent by a convicted prisoner in Scotland in “purposeful activity” (eg, employment and education) was 20 h/week, influenced by factors such as the prison regime, availability of spaces, and prisoner–staff ratios.^20^ Being able to find meaningful ways of passing time in prison may impact on people’s physical and psychological health and use of substances such as tobacco, e-cigarettes, and illicit drugs.^21^

Implementation of smokefree policy has distinct implications in this setting. Prisons are both home for those in custody and a staff workplace, and smokers cannot leave the perimeter, or move freely through parts of the prison. As reported elsewhere,^22^ support for smokefree policy was lower among people in custody than prison staff, reflecting concerns about the legitimacy and fairness of smokefree rules and worries about potential adverse consequences. At the same time, perspectives on smokefree prison policy among people in custody can be complex: potential benefits for prisoner (and staff) groups are often acknowledged and motivations to quit smoking are high in the prison population,^4^ as for other smokers. This study explores initial views and experiences of vaping in this specific policy and institutional context, when vapes were new and removal of tobacco was imminent.

## Results

### Participants

Almost all participants had started smoking in or before adolescence (21 were current smokers, six were former smokers [who had essentially switched to e-cigarettes in prison, see below], and one was neither smoking nor vaping), prior to their current custodial sentence. Interest in smoking cessation varied among participants; however, few expressed no or little interest in quitting smoking eventually. While most had made at least one previous cessation attempt, some had only ever stopped smoking for circumstantial reasons (eg, while in police custody or hospital).

At the time of interview, almost all had their own rechargeable e-cigarette(s) with pre-filled e-liquids, but patterns of e-cigarette use varied: six were exclusively or almost exclusively vaping (“exclusive-vaper”); 17 were vaping and (mostly daily) smoking (“dual-user”); and five had tried e-cigarettes in prison but were not currently vaping (“former-vaper”). Reasons for not vaping varied, including not finding vaping satisfactory, wanting to only smoke tobacco at that time, and having vaped out of curiosity. Ten participants had previously vaped outside of prison; 13 had not, including some who entered prison before e-cigarettes became widely available in the United Kingdom. The vaping history of five participants is unknown.

### Support for E-cigarettes in Prison and Reasons for Use

Participants expressed strong support for e-cigarettes becoming available before smokefree prison policy. They described their symbolic and practical value in helping people in custody to manage without tobacco in the near future. E-cigarettes were viewed as a welcome and unexpected gesture of support by the prison service and a “quid pro quo” in the light of the removal of tobacco, illustrated by a participant (Dual-user, R04.03) who said: “at least they gave us something in return, at least they’re not just whipping it [tobacco] away….” E-cigarettes were also seen to be of practical benefit for smokers who did *not* want to abstain or in providing those interested in smoking cessation with another means of doing so.

Some participants first tried single-use e-cigarettes when these were introduced during earlier preparations for smokefree policy. Their largely negative experiences of these devices did not appear to deter them from later trying rechargeable e-cigarettes in prison. Uptake of rechargeable e-cigarettes was strongly influenced by the imminent smokefree policy and related to three main themes.

First, smokers were aware they had to find ways of quitting or abstaining from smoking because of forthcoming tobacco restrictions: “we’ve no choice...we’ve just got to, and that’s it” (Dual-user, ST01.02). Some smokers were also motivated to try vaping because of the potential health or financial benefits of quitting or cutting down on smoking. As the two contrasting quotes below illustrate, smokers could present vaping as a means of retaining control over their smoking behavior or a loss of freedom in the context of the smokefree policy:

Dual-user, LT06.04: “A lot of guys have switched over [to vaping] already, you know, so that they’re not getting told to do it. They’ve done it off their own backs.”

Dual-user, LT01.04: “…they’re…taking my snout [tobacco] off us. That’s what made me try it [e-cigarettes].”

Second, e-cigarette “starter packs” were being distributed on an interim basis to eligible declared adult smokers, meaning many could try vaping for free in preparation for the removal of tobacco. The distribution of starter packs was perceived to have helped to ensure that “everyone got the chance” (Exclusive-vaper, ST04.01) to prepare for the ban and encouraged some to try e-cigarettes in advance of the smokefree rules:

Exclusive-vaper, ST-04.01: “…it was a new thing coming into the jail, and we got them for free. So it’s sitting there, so it’s just a case of ‘I’ll use it and see what it’s like’, we got oils [e-liquids] with it too for free.”

Third, there was considerable curiosity about the introduction of a novel product (rechargeable e-cigarettes) in an environment in which there is considerably less variety and choice of consumer products than in wider society:

Dual-user, LT04.06: “…when we all got our vapes, I was buzzing ‘cause I’ve been in here for two year…I was like, ‘oh it’s something new’.”

### Initial Experiences of Vaping in Preparation for Smokefree Policy

As rechargeable e-cigarettes had only been on sale in prisons for a month or two, some participants were still learning to use their devices. At this time, patterns of vaping varied, with some reporting near continuous use of e-cigarettes, while others were able to “pick up and put down” (Exclusive-vaper, R06.01) e-cigarettes as needed. Current vapers described making progress in reducing or, less frequently, stopping tobacco use: some had exceeded their own expectations and so felt more confident they would manage without tobacco in the future:

Dual-user, LT06.04: “I’ve gone a whole day just on the vapes.

**Interviewer: And how has that gone?**

LT06.04: I was quite surprised.

**Interviewer: In a good way or a bad way?**

LT 06.04: In a good way because now I know I can do it and it’s not going to be as sore as what I thought it was. You know, just a complete withdrawal.”

One perspective was that the resemblance of vaping to some of the actions of smoking was beneficial for smoking abstinence. One (Exclusive-vaper, ST03.04) commented: “…it’s obvious that they [product manufacturers] have tried to fill…all the steps… lifting something to your mouth, puffing and blowing out smoke [vapour].” He continued: “certainly with the brand that I’m using it does fulfill that need for nicotine and like I say, stressful times, and after a meal, and things like that.”

Others spoke in strong terms about encountering difficulties in managing nicotine cravings by vaping. They felt the e-cigarettes they had tried in prison had not always met their needs in speed of nicotine delivery or desired strength of “hit”:

Exclusive-vaper, LT6.05: “As it is now, I can’t even get a vape strong enough to satisfy, without me sitting puff, puff, puffing, you know. And that’s what I’m doing, you know.”

In addition to the potential utility of e-cigarettes in helping with smoking abstinence/cessation in the future, participants also commented on the pleasure, satisfaction, and (initial) sense of excitement that vaping was providing some people in custody. Flavored e-liquids were cited as a potentially important part of the appeal of vaping, reflected in reportedly a good deal of interest in, and experimentation with, flavored e-liquids among people in custody at that time.

Former-user, ST04.05: “…berrymint you can just keep smoking [vaping], because they taste nice, so you enjoy smoking [vaping]…”

### Initial Views on Product Choice, Cost, Safety, and Long-Term Use

Participant views on issues relating to e-cigarette use in the prison population more specifically (product choice and cost) and more generally (safety and long-term use) are explored below.

#### Product Choice

Some participants reported dissatisfaction with the choice of rechargeable e-cigarettes on sale in prison, which was very limited compared to wider society. Some strongly expressed requests for a greater range of e-liquids, including higher strengths for heavy smokers and lower for those who wanted to reduce their nicotine intake or quit vaping. There were also requests for a greater range of flavors and strengths of e-liquids and more brands of rechargeable e-cigarettes (including a more powerful device) via the canteen:

Exclusive-user, sentence length unknown 01.06: “… some people might see them as a substitute, and they might keep vaping, but I see it as a way to try and stop [nicotine]… I think if you can cut down, I don’t know if there would be one lower than that [lowest strength (12mg) of e-liquid sold in prison], and just gradually cut down, and get rid of nicotine altogether.”

#### Cost

The cost of items sold in the canteen matters a great deal to people in custody who describe needing to “watch what you spend in here” (Dual-user, ST01.02), due to factors such as weekly spending limits and low wages. In a context in which most participants were temporarily dual-using e-cigarettes and tobacco, some were very positive about actual or potential cost savings from switching to vaping:

Dual-user, LT01.01: “[e-cigarettes] should be more affordable than tobacco… ‘cause we don’t get a lot of wages in here, £11 and things like that…”

Participants described how savings could be used to meet other needs, eg, buying food/snack items, tea/coffee, and phone credit to maintain family contact or building reserves to use the following liberation.

Dual-user, ST01.03: “It’s good and having extra money on the phone, I can speak to my family a bit more and my friends and stuff. It’s a lot better.”

However, others expressed concerns about the long-term affordability of vaping, foreseeing possible hardship for certain groups (especially for individuals not in receipt of external financial assistance), and high nicotine dependence in the prison population. Indeed, some were already reporting that e-liquids were not lasting long enough or thought this could a problem in the future, potentially making it hard for some to manage supplies between (typically) weekly canteen deliveries.

Former-vaper, LT02.01: “….a lot of people [in prison] chain smoke, you know. They are going to be the hardest hit….They don’t last long…If you keep smoking [vaping], taking the draws, it’s away in no time.”

Dual-user, LT04.02: “I get through one of those [e-liquids] a day. So that’s lasting me three days. You’ve got seven days before you get your next canteen, so you’re four days with none.”

#### Perceived Short- and Long-Term Safety of E-cigarettes

While on the one hand some individuals reported short-term health improvements which they associated with smoking reduction/abstinence, on the other hand confusion, uncertainty, and skepticism were often expressed about the safety of vaping. Some questioned the logic of replacing one harmful product (tobacco) with another (e-cigarettes) which they thought may also carry health risks: “…no point in stopping one thing that’s bad for you and doing another thing that’s bad for you” (Dual-user, LT01.01). Health concerns were often balanced by the perceived need for people in custody to have a range of options, including guaranteed long-term availability of e-cigarettes, to help them manage mandated smoking abstinence. Some wanted more information on vaping and its effects and expressed a desire to be kept up-to-date as new evidence emerged.

#### Temporary Versus Long-Term Use of E-cigarettes

Participants expressed uncertainty about whether they would vape on a temporary or long-term basis, partly because, in the words of one (Dual-user, LT01.05), some people in custody were not thinking “…that far ahead yet” as they were yet to experience smokefree policy or were still in the process of making up their minds about e-cigarettes. Several explanations were given for why some people in custody might eventually want to stop vaping, including not wanting to “swap” one form of addiction for another, to save money or for health reasons. Participants were generally unsure about the potential addictiveness of e-cigarettes and thus any future difficulty in being able to give up vaping. Some participants expressed surprise or discomfort about the amount/frequency of their current vaping, commenting on vaping as one limited means of enjoyment or passing time when locked in cells, reflecting previously entrenched tobacco smoking practices in prison. This could be seen as a virtue or hazard of e-cigarettes, depending on an individual’s attitude:

Dual-user, R03.03: “[T]hat is what kind of bothers me sometimes because there are [times] when I lift it [e-cigarette] up, I do enjoy it, do you know what I mean, and sometimes I don’t want to put it back down.”

## Discussion

It is recognized that smokers should have access to options for managing nicotine dependence or withdrawing from nicotine as part of tobacco cessation,^23^ particularly in the lead up to, and following, the creation of smokefree settings. In this study, conducted in the near-unique circumstances of introducing rechargeable e-cigarettes into a prison system shortly before mandated smoking abstinence, the use of e-cigarettes was strongly influenced by participants’ need to prepare for day-to-day life without tobacco. Free provision of e-cigarette starter packs to eligible smokers appears to have facilitated experimentation with vaping in preparation for smokefree policy. This may have made it easier for some people to adjust during a challenging transition period, potentially reducing financial inequities in access to products for those wishing to vape rather than use (free) NRT. This study provides a snapshot of vaping in prison prior to vaping norms, habits, and practices in the prison population becoming established. However, our findings indicate some differences that may potentially emerge in the future between people in custody and other (vulnerable) user groups. These relate, for example, to distinct social and environmental influences on vaping patterns, including long periods locked in cells and restrictions on where vaping is permitted; and challenges in meeting individual product preferences given the limited range of items that are sold in prison, infrequent purchasing opportunities (usually weekly) and rules in prisons on receiving/earning and spending money.

Another important potential difference between those in custody and other (vulnerable) user groups is that the role of e-cigarettes within smokefree prisons is less about harm reduction (as conventionally understood) and more about supporting people to manage in a situation of enforced abstinence and reducing other potential adverse unintended harms more broadly defined of smokefree policies. The potential role of e-cigarettes in reducing tobacco-related harms (by minimizing the risk of return to smoking) in people leaving smokefree prisons is an important area of future study.

By contrast, other elements of participants’ experiences of vaping in prison are consistent with evidence on vaping in the wider population. Parallels with published work on e-cigarettes include evidence of the potential role of flavored e-liquids in increasing the appeal of e-cigarettes (including for smoking cessation)^24–26^; suggestions that for some smokers the appeal of e-cigarettes is linked to physiological, behavioral, and/or lifestyle similarities of vaping to smoking^27–29^; and reports that some individuals struggle to manage withdrawal symptoms by vaping.^30,31^ There are also some similarities to the literature on other vulnerable, potentially intersecting, populations (eg, those with severe mental health problems using health services and homeless people) who experience some common barriers to tobacco cessation (eg, high stress levels, lack of other “comforts,” boredom, smoking norms).^32^ Areas of overlap include the role of smokefree policy as a facilitator for e-cigarette use,^33^ particularly for those who are unable to quit or wish to replicate some psychological functions of smoking, and the importance of cost implications of switching to vaping.^33^

A key strength of this study is that it provides timely insight into early views and experiences of vaping among a diverse group of individuals (by sex, remanded/convicted status, sentence length, and prison environment) when introduced across the prison system as part of smokefree policy. We are not aware of any published studies of vaping in prison and believe the data are unique internationally; our research thus contributes vital evidence to a sparse knowledge base. The findings have helped to inform and support policy and service development in smokefree prisons in Scotland^34^ and could support the development of policies in prisons in other jurisdictions with similar regulations on e-cigarettes as the United Kingdom. Insights from this, and future work, might also be relevant for other smokefree settings for vulnerable groups (eg, people with severe mental health problems), depending on the degree of similarity in terms of restrictions on movement/duration of stay, availability of distraction activities/ways of occupying time, and user incomes and (in)ability to access a range of products.

We also note some limitations. These include the risk of sample bias due to the use of “gatekeepers” for recruitment and interviews being conducted in a subset (6/15) of Scottish prisons, and any self-selection bias intrinsic to participants being fully able to choose whether to participate in interviews. Efforts were made to reduce selection bias by careful explanation of the study to “gatekeepers” and potential participants. Another limitation is that we did not collect detailed information on participant smoking habits and history, partly due to constraints and challenges in timings within the prison regime, and the burden on participants and staff. Importantly, the data were collected at a particular moment in time immediately leading up to the implementation of smokefree prison policy: it is likely perceptions and experiences of vaping will evolve in this context as policy embeds.

In smokefree prisons (and similar settings) that permit vaping, there is a continuing need for public health interventions, to help inform people in custody about evolving evidence of relative risks of different nicotine products and to maximize potential benefits of smokefree policy. Smokefree prisons also require nicotine management services that can work effectively with diverse service users, including those who vape. These could help reduce potential adverse consequences of vaping through the provision of information and assistance to individuals who decide to quit vaping while in prison. Health care providers might face challenges in adapting conventional cessation services in response to e-cigarettes. Vaping remains contentious within public health, the knowledge base on supporting individuals to reduce or stop vaping is scant, and there are many competing demands on health care resources. There are clear benefits in ensuring people in custody, families, and frontline staff are involved in the (re)design of nicotine management services and that services are delivered within a whole-prison health-promoting environment.

Given e-cigarettes are a relatively new technology, their use in prison should be monitored longer term to enable early identification and mitigation of any unintended consequences, such as using devices to consume other substances. A subsequent phase of this study will provide updated evidence on experiences of vaping among people in custody, and on prison staff views about (intended and unintended) consequences, based on interviews conducted 6 months after smokefree policy was enacted.

In conclusion, our findings suggest the potential benefits of e-cigarettes as one means of supporting the removal of tobacco in a population with very high smoking rates. They also point to possible challenges for vaping in prisons and similar smokefree settings. There is a need for ongoing measures to maximize the health benefits of smokefree policy, and for further research on vaping in situations of enforced abstinence from smoking, particularly where restricted movement, product purchases, and opportunities to vape distinguish this group from e-cigarette users in the general population and more particularly other vulnerable groups.

## Supplementary Material

A Contributorship Form detailing each author’s specific involvement with this content, as well as any supplementary data, are available online at https://academic.oup.com/ntr.

ntaa088_suppl_Supplementary_Taxonomy
